# Event-Related Potentials in Women on the Pill: Neural Correlates of Positive and Erotic Stimulus Processing in Oral Contraceptive Users

**DOI:** 10.3389/fnins.2021.798823

**Published:** 2022-01-04

**Authors:** Norina M. Schmidt, Juergen Hennig, Aisha J. L. Munk

**Affiliations:** Department of Differential and Biological Psychology, University of Giessen, Giessen, Germany

**Keywords:** oral contraceptives (OCs), event-related potentials (ERP), neural reactivity, emotional processing, early posterior negativity (EPN), late positive potential (LPP), gonadal steroids, subjective stimulus evaluations

## Abstract

**Background/Aims:** Exposure toward positive emotional cues with – and without – reproductive significance plays a crucial role in daily life and regarding well-being as well as mental health. While possible adverse effects of oral contraceptive (OC) use on female mental and sexual health are widely discussed, neural processing of positive emotional stimuli has not been systematically investigated in association with OC use. Considering reported effects on mood, well-being and sexual function, and proposed associations with depression, it was hypothesized that OC users showed reduced neural reactivity toward positive and erotic emotional stimuli during early as well as later stages of emotional processing and also rated these stimuli as less pleasant and less arousing compared to naturally cycling (NC) women.

**Method:** Sixty-two female subjects (29 NC and 33 OC) were assessed at three time points across the natural menstrual cycle and corresponding time points of the OC regimen. Early (early posterior negativity, EPN) and late (late positive potential, LPP) event-related potentials in reaction to positive, erotic and neutral stimuli were collected during an Emotional Picture Stroop Paradigm (EPSP). At each appointment, subjects provided saliva samples for analysis of gonadal steroid concentration. Valence and arousal ratings were collected at the last appointment.

**Results:** Oral contraceptive users had significantly lower endogenous estradiol and progesterone concentrations compared to NC women. No significant group differences in either subjective stimulus evaluations or neural reactivity toward positive and erotic emotional stimuli were observed. For the OC group, LPP amplitudes in reaction to erotic vs. neutral pictures differed significantly between measurement times across the OC regimen.

**Discussion:** In this study, no evidence regarding alterations of neural reactivity toward positive and erotic stimuli in OC users compared to NC was found. Possible confounding factors and lines for future research are elaborated and discussed.

## Introduction

The majority of previous research regarding effects of oral contraception (OC) has been focusing on its physiological aspects (Frye, 2006) and side effects like cancer risk (Gierisch et al., 2013), or thromboembolic events (Oedingen et al., 2018). Psychophysiological aspects of OC use have been studied less systematically (Pletzer and Kerschbaum, 2014). OCs might, however, be shaping mind and behavior via their influence on the endocrine as well as the central nervous system. So called combined OCs (COCs) are the most commonly used ones and contain 20–35 μg ethinyl-estradiol (EE) and varying progestin components (Dhont, 2010; Liang et al., 2012). These synthetic steroids unfold negative feedback effects on the hypothalamus-pituitary-gonadal (HPG-) axis. During intake they consequently reduce serum levels and cyclical fluctuations of neurosteroid precursors (i.e., progesterone, pregnenolone, EE, and testosterone) as well as neurosteroids (i.e., allopregnanolone and allotetrahydrodeoxycorticosterone) (Rapkin et al., 2006; Zimmerman et al., 2014). Levels of endogenous gonadal steroids also stay suppressed during the 7 days OC break, even though some residual ovarian activity can occur (van Heusden and Fauser, 1999). Neurosteroids impact activity as well as organization of the nervous system due to their influence on synaptic transmission, myelination, apoptosis, and dendritic spine plasticity via genomic and non-genomic pathways (Del Río et al., 2018). Accordingly, neuroanatomical alterations regarding gray and white matter density and/or volume have been reported in OC users – typically in areas of the limbic system (Brønnick et al., 2020; Taylor et al., 2021) which is known for its key role in emotional processing (Frühholz et al., 2014). Using structural magnetic resonance imaging (MRI) data, Hertel et al. (2017) observed lower hippocampal volume and hypothalamic-pituitary-adrenal (HPA-) axis alterations (i.e., elevated baseline cortisol levels) consistent with chronic stress in OC users. Lisofsky et al. (2016) reported reduced gray matter volume in OC users’ left amygdala and anterior parahippocampal gyrus. In contrast, Sharma et al. (2020b) reported greater white matter volume in OC users’ right amygdala, left parahippocampal gyrus and left hippocampus while not observing any gray matter differences in these regions. Regarding brain connectivity, Petersen et al. (2014, 2015) reported reduced cortical thickness in parts of the default and salience network in OC users and altered resting state functional connectivity in the default and executive control network, which are involved in reward processing and evaluation of internal and external stimuli. Correspondingly, differences between OC users and naturally cycling (NC) women have been reported in a variety of psychophysiological functions, including stress responsivity, fear conditioning, cognition and socio-emotional behaviors such as emotional processing (Warren et al., 2014; Montoya and Bos, 2017; Lewis et al., 2019).

Due to previously reported associations of OC use and depression (Skovlund et al., 2017; Wit et al., 2020) as well as a reduction of general well-being and its subdomains positive well-being, self-control and vitality (Zethraeus et al., 2017), most of the existing neuropsychological OC research has been investigating processing of negative stimuli (Brønnick et al., 2020), or adverse mood effects (Schaffir et al., 2016). Using functional magnetic resonance imaging (fMRI), Petersen and Cahill (2015) reported reduced bilateral amygdala reactivity toward negative visual stimuli in OC users. Gingnell et al. (2013) additionally collected daily symptom ratings and observed lower emotional reactivity toward negative facial expressions in OC users in the left insula, left middle frontal gyrus and the bilateral inferior frontal gyri along adverse mood effects such as depressed mood, mood swings and fatigue after one cycle of OC treatment in a placebo controlled, double-blind randomized controlled trial (RCT) (Gingnell et al., 2013). While the authors studied women who had previously experienced negative mood symptoms during OC intake (Gingnell et al., 2013), results on general mood effects of OC use are still inconclusive (Schaffir et al., 2016). Some studies report mood worsening – especially during the hormone-free phase (Lundin et al., 2017). Others indicate beneficial effects including mood improvement, higher mood stability and a decrease in negative premenstrual affect (Oinonen and Mazmanian, 2002; Jarva and Oinonen, 2007; Hamstra et al., 2017). In contrast, positive emotional states and processing of positive stimuli under OC use are examined only very rarely. These factors are, however, highly relevant for well-being and mental health (Alexander et al., 2021). Regarding depression, numerous studies reported a reduction of positive affect (Gençöz, 2002; Wichers et al., 2007; Horner et al., 2014) and stressed the importance of targeting low positive affect specifically in anti-depressive treatments (Nutt et al., 2007; Werner-Seidler et al., 2013; Jonge et al., 2017). Depressive subjects also show reduced neural reactivity toward positive emotional stimuli which might explain the occurrence of depressive symptoms, such as loss of interest and anhedonia (Shestyuk et al., 2005; Epstein et al., 2006). In one of the few studies on this topic regarding OC use, Jarva and Oinonen (2007) observed blunted self-reported emotional reactivity following positive mood inductions while emotional reactivity following negative mood inductions was unchanged. The authors argued that OC’s mood stabilizing effect, accompanied by reduced positive emotional reactivity, might resemble depressive states reported by some users. Further research in this field is highly necessary, especially since perceived adverse influences of OCs on mental health are a common reason for discontinuation of their use (Sanders et al., 2001). The same is true for deteriorations in *female sexual function* (Sanders et al., 2001) which refers to the entirety of psychological and physiological aspects of the female sexual response (Basson, 2008). Some authors argue that OCs should have positive effects on female sexuality as their conception-suppressing effect ensures greater sexual freedom and reduces pregnancy-related anxieties during sexual intercourse (Burrows et al., 2012). Others have reported lower female sexual function scores in users of oral vs. other forms of contraception (Wallwiener et al., 2010, 2015), or reduced scores in its subdomains including arousal, pleasure, orgasm and lubrication (Smith et al., 2014). Results are so far inconclusive and differ greatly with respect to study design (Davis and Castaño, 2004; Pastor et al., 2013). Still, RCTs and meta-analytic reviews suggest that the female sexual function subdomain *desire* might be affected by OC use (Zethraeus et al., 2016; Huang et al., 2020). Altered processing of erotic stimuli has been reported in females suffering from hyposexual desire disorder (Bianchi-Demicheli et al., 2011; Woodard et al., 2013). Regarding OC use, Abler et al. (2013) compared different stages of erotic stimulus processing in 12 OC users and 12 NC women. They reported reduced brain activity during anticipation of erotic stimuli. During viewing of erotic pictures and videos, no consistent significant differences were observed between both groups. OC and NC subjects did also not differ in arousal ratings of erotic stimuli. While this is – to be best of our knowledge – the only study that explicitly compared erotic stimulus processing in NC and OC women, erotic stimulus processing has more often been examined in association with the natural menstrual cycle (MC) using fMRI (Gizewski et al., 2006; Zhu et al., 2010) as well as event-related potential (ERP) techniques (Krug et al., 2000; Munk et al., 2018, 2020).

The use of ERPs has important advantages because their high temporal resolution allows to assess immediate vs. prolonged neural reactions associated with attending to and processing of motivationally salient stimuli (Brown et al., 2013). Compared to commonly conducted (f)MRI investigations, ERP studies also have fewer exclusion criteria (e.g., dental braces, retainers, bone plates and screws, implants) (McLoughlin et al., 2014) allowing greater and more integrative samples. In neuroendocrine research, which is already characterized by numerous exclusion criteria (i.e., absence of any physical or psychological illness, no medication intake, regular menstrual cycles), this is a huge benefit regarding representativeness and generalizability of results. Most ERP studies regarding MC effects focused on the late positive potential (LPP) – an EPR component most prominent on centro-parietal electrode sites. It starts around 400 ms after stimulus onset and can last for several hundred ms. The LPP is sensitive to emotional content with typically higher amplitudes toward emotional vs. neutral stimuli (Schupp et al., 2004, 2006; Hajcak and Olvet, 2008). It is therefore suggested to reflect prolonged and facilitated attention toward motivationally salient emotional stimuli and their processing (Foti et al., 2009; Hajcak et al., 2012). Regarding the MC, Krug et al. (2000) observed higher LPP amplitudes toward erotic pictures around ovulation when these stimuli are highly relevant for fertility and reproduction. Similarly, Munk et al. (2018, 2020) reported that LPP amplitudes toward erotic vs. neutral stimuli were associated with estradiol concentration which typically peaks during ovulation. As OCs suppress ovulation and inhibit conception, they might also reduce motivational salience of erotic stimuli. Furthermore, the LPP has been associated with activity of the dopaminergic reward system (Cuthbert et al., 2000; Munk et al., 2016). Activity of this system is modulated by estradiol and testosterone, both of which are downregulated by OC intake (Rapkin et al., 2006; Zimmerman et al., 2014; Montoya and Bos, 2017). Recently, another ERP component has been suggested to be especially sensitive to erotic content– the early posterior negativity (EPN) (Frank and Sabatinelli, 2019; Farkas et al., 2020). The EPN is defined as a relative negativity at temporo-occipital electrode sites that is also sensitive to emotional content but occurs earlier than the LPP at approximately 150–300 ms after stimulus onset (Schupp et al., 2004, 2006; Hajcak et al., 2012). It is therefore associated with earlier and broader stimulus distinction compared to the LPP (Weinberg and Hajcak, 2010; Farkas et al., 2020). To the best of our knowledge, no study so far assessed the EPN in association with MC or OC effects. However, gender differences have been observed with reduced EPN modulation in women vs. men (Weinberg and Hajcak, 2010), indicating some involvement of sex steroids. Furthermore, Farkas et al. (2020) observed greater EPN negativity in reaction to erotic and nudist scenes compared to other pleasant and unpleasant visual stimuli and interpreted this as a marker of heightened attention for sexual opportunities – a process that might be modulated by OC use.

To differentiate between earlier vs. later stages of emotional processing might be relevant in association with OC use, as a recent ERP study provided initial evidence for differential effects of OC use at different processing stages (Monciunskaite et al., 2019). In this only ERP study on the effects of OCs to date, the authors used a passive viewing task to examine processing of (highly) unpleasant and pleasant (i.e., erotic) as well as neutral social visual stimuli derived from the International Affective Picture System (IAPS). Monciunskaite et al. (2019) reported significantly lower LPP amplitudes and global field power (GFP) in OC users compared to NC subjects in reaction to all picture categories. When comparing emotional-neutral difference scores in the LPP, however, the only significant finding was an attenuated unpleasant-neutral difference in OC users. Furthermore, OC users showed higher GFP (trend level) and significantly higher parieto-occipital activity compared to NC women in an earlier time window (<350 ms), pointing toward differential effects of OC use on early vs. later stages of emotional processing (Monciunskaite et al., 2019). An effect of emotion regulation strategies such as reappraisal on neural reactivity was discussed, yet, no subjective stimulus evaluations were assessed to support this hypothesis (Monciunskaite et al., 2019). Previous results on subjective stimulus evaluations in OC users have revealed inconsistent results. Whereas some studies reported no differences regarding positive, neutral, negative (Armbruster et al., 2017) nor erotic (Abler et al., 2013) stimuli, others reported higher valence ratings in reaction to emotional- and lower arousal ratings in reaction to neutral stimuli in users of different forms of hormonal contraceptives (HCs) (Spalek et al., 2019).

Another important limitation of Monciunskaite et al.’s (2019) – and also other studies on OC use – is that OC users were assessed only once, namely during the active period of the OC regimen. Yet, most adverse effects of OC use are being reported during the 7 days break (Sulak, 2000; Kelly et al., 2010). Studies that included measurements during the OC free week reported differences in brain connectivity and emotional processing between active and OC free periods. Nasseri et al. (2020) noticed higher amygdala-ventromedial prefrontal cortex (vmPFC) coupling in the left hemisphere after stress exposure during the active vs. OC free period and interpreted this in terms of better emotion regulation abilities during OC intake. Similarly, Radke and Derntl (2016) reported higher accuracy in an affective responsiveness task during the active vs. OC free period of the regimen. As Herrera et al. (2020) point out, consideration of OC regimen is also important to disentangle effects associated with either high exogenous hormone intake or reduced endogenous hormone production.

Therefore, the current study examined early vs. prolonged stages of positive and erotic stimulus processing in OC users across different time points of the OC regimen. A within-subjects repeated-measurements design was chosen in order to overcome limitations of frequently used between-subjects designs, in which groups of women in different phases of the OC regimen/MC have been compared. The most important aim was to elucidate overall differences between OC using and NC women as well as OC regimen – rather than MC related – effects on emotional processing.

Regarding subjective stimulus evaluations as well as neural reactivity toward emotional stimuli, following hypotheses were investigated:

(1) Higher valence and arousal ratings as well as EPN modulation and LPP amplitudes in reaction to emotional vs. neutral stimuli (Manipulation Check).
(2) Lower valence and arousal ratings as well as EPN modulation and LPP amplitudes in OC users.
(3) A modulation of ERPs by OC regimen period (active vs. OC free).

## Materials and Methods

### Participants

Women were recruited at the University of Giessen via circular emails and flyers and screened for fulfillment of inclusion criteria using a telephone-based interview, which was conducted after subjects were found eligible using a self-designed online screening questionnaire. Inclusion criteria were: age between 18 and 35 years, nulliparous, absence of any physical or psychological illness, no intake of medication or drugs (incl. tobacco) influencing the central nervous- or endocrine system, right-handedness, body-mass index (BMI) ≥ 18 ≤ 26 kg/m^2^ and normal or corrected-to-normal vision. Parous women were excluded as several studies indicate endocrine differences between nulliparous and parous women (e.g., Bernstein et al., 1985; Hill et al., 1986; Musey et al., 1987; Barrett et al., 2014). Left-handed subjects were excluded to avoid lateralization effects (Sainburg, 2014). 87 native German-speaking females started participation in the study. Testing could not be completed with 21 women (*N* = 15: contact restrictions during the coronavirus pandemic; *N* = 3: no detectable ovulation, *N* = 3: difficulties with scheduling), leaving a sample of 66 (30 NC and 36 OC) women. NC women reported a regular MC duration of 26–30 days (mean ± SD: 27.97 ± 1.22). 22 (73%) stated to have previously used hormonal contraceptives. While a hormone-free interval of at least 6 months was required for participation, mean time since discontinuation was 49.14 months (*SD* = 30.99, range: 7–177 months). The OC group consisted of women using combined monophasic OCs with an EE-dosage <50 μg for at least 6 months. An OC regimen of 21/7 (21 days OCs and 7 days OC free) was required for participation. Mean duration of OC intake was 58.18 months (*SD* = 31.12). 25 subjects used OCs that contained androgenic progestins; eleven subjects used OCs containing anti-androgenic progestins. Androgenicity of progestin components was classified according to Wiegratz and Thaler (2011). One subject in the OC group was excluded from all further analyses as she reported – after completion of the study – that she was using an OC (“Zoely”) which contains estradiol instead of EE and is taken in a 24/4 regimen. An overview of EE-dosages and progestin components can be found in the Supplementary Material.

Subjects participated in exchange for a monetary compensation of 10€/h or research participation credit. Written informed consent was obtained. The study was conducted in accordance with the declaration of Helsinki and was approved by the local ethics committee of the University of Giessen, Department of Psychology (application number: 2018-0022).

### Study Design

**FIGURE 1:**
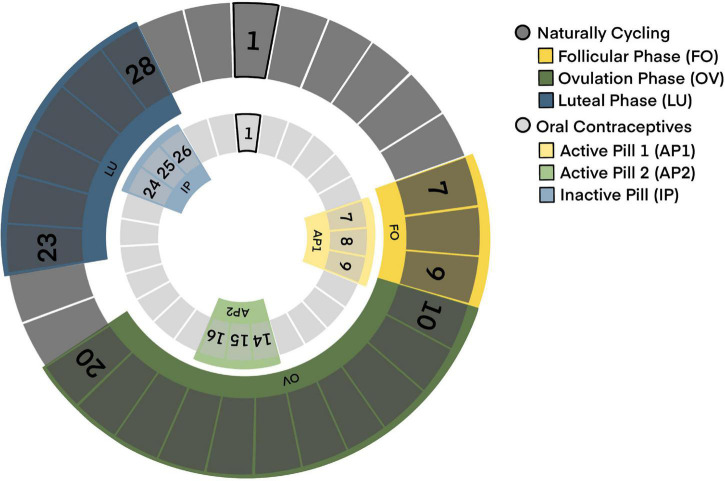
Testing dates for naturally cycling and oral contraceptive using women.

This study was conducted as a counter-balanced repeated-measurements design. Each woman attended three testing sessions across one or two consecutive MCs or OC regimen. Females in the OC group were tested on days 7–9 (mean ± SD: 7.97 ± 0.82) and days 14–16 (mean ± SD: 15.03 ± 0.79) of their OC blister (measurement times AP1 and AP2, respectively) as well as during their OC free week (measurement time IP), i.e., days 24–26 after starting a new OC blister (mean ± SD: 24.91 ± 0.70). Measurements during the OC free week corresponded to the first 2 days of the withdrawal bleeding to ensure that women were in a hormone-withdrawal state. Testing dates of the NC group were adapted to the women’s average MC length. Measurements during follicular phase (FO) were conducted on days 7–9 (mean ± SD: 7.78 ± 0.63). Measurements during luteal phase (LU) were terminated according to the following formula: average MC length – 2/3 days. Consequently, they were conducted on days 23–28 (mean ± SD: 25.57 ± 1.25). For a correct termination of measurements during ovulation (OV), NC women were provided with LH-tests (Femometer LH Ovulation Rapid Test Strip; sensitivity 25 miu/ml; Hangzhou Clongene Biotech Co., Ltd.; Hong Kong) that they were supposed to use for seven consecutive days around their predicted ovulation. After announcing a positive LH-test result, subjects were assessed within 24 h (mean ± SD: 8.79 ± 7.03). OV measurements were conducted between days 10–20 (mean ± SD: 14.90 ± 2.44). To avoid sequence effects, phase of first measurement was counter-balanced. 11/16 women started during FO/AP1, 7/14 subjects during OV/AP2 and 12/5 subjects during LU/IP. It was aimed to schedule all three testing appointments at approximately the same time of the day (morning, noon, and afternoon) to account for the circadian rhythmicity in gonadal steroid secretion. To ensure similar between-session intervals for the NC and OC group, OC users were tested twice during their active OC period with the second time point corresponding to ovulation in NC women. Testing dates for both groups are illustrated in Figure 1.

### Emotional Picture Stroop Paradigm

To assess neural reactivity toward emotional stimuli, an Emotional Picture Stroop Paradigm (EPSP) was conducted. EPSPs were previously successfully used as an implicit measurement tool regarding sexual interest (Ciardha and Gormley, 2009; Munk et al., 2020). The combination of EPSPs and ERP collection is, moreover, advantageous in assessing selective attention and has proven as a valid instrument in emotion research (Thomas et al., 2007; Bertsch et al., 2009; Franken et al., 2009). Based on previous research (Munk et al., 2020), three main categories were included: erotic stimuli included couples in underwear and erotic poses (couple erotic) as well as lightly dressed males (male erotic) and females (female erotic). Positive stimuli included single smiling individuals of both sexes; neutral stimuli consisted of couples (couple neutral) and single individuals (person neutral) in neutral poses and with neutral facial expressions, and trees as a non-social neutral stimulus category. In order to validate picture selection regarding valence and arousal, an online pilot-study in *n* = 134 women (mean age ± SD: 25.57 ± 8.99) had been conducted before. Women rated 84 preselected pictures (12 per subcategory) on valence and arousal. Pictures were retrieved from www.shutterstock.com and consecutively gray-scaled. Eight pictures per subcategory were chosen for the main study based on the following criteria: High valence as well as arousal ratings for each of the erotic subcategories, high valence and medium arousal ratings for the positive category and medium valence along low arousal ratings for the neutral categories. Higher arousal ratings were obtained for the erotic vs. positive and both emotional vs. the neutral category. Valence ratings were higher for the positive vs. erotic and both emotional vs. the neutral category.

**FIGURE 2:**
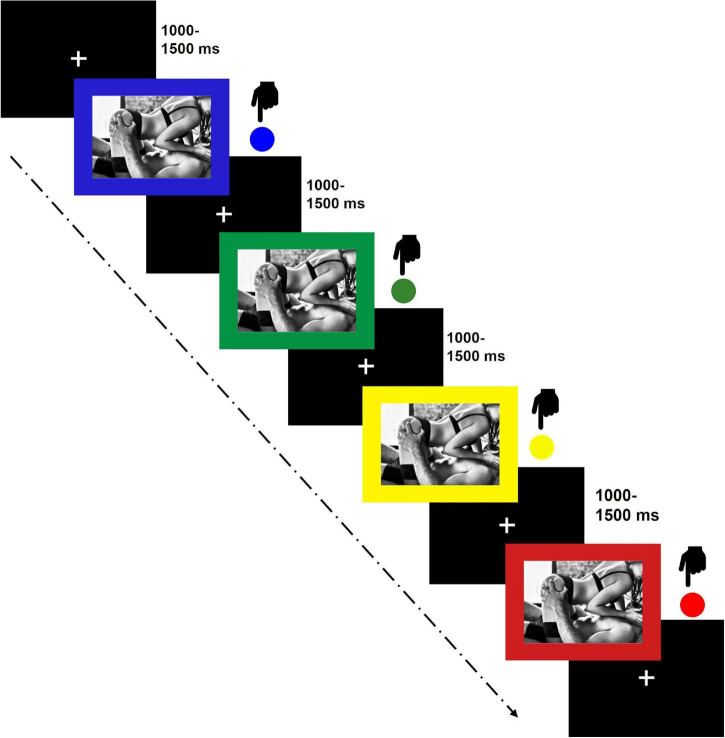
Emotional Picture Stroop Paradigm (EPSP).

During the EPSP, gray-scaled pictures were presented in a size of 640 pixels × 480 pixels on a black background. Subjects were seated in a comfortable chair in a dimly lit room at a 60 cm distance to the screen of 24′′. They were instructed to indicate the picture’s frame color (blue, green, red, and yellow) by pressing the respective button as fast and as accurately as possible on a response pad (MilliKey™ MH-5; Lab Hackers Research Equipment, Halifax, Canada). Picture presentation lasted until subjects executed a response. An inter-trial interval (white fixation cross) with a random duration of 1,000–1,500 ms (mean 1,250) was presented between trials (Figure 2). Each picture was presented twice in each frame color resulting in 64 trials per subcategory and a total of 448 trials divided into four blocks of 112 trials each. Order of trials was randomized. Between blocks, a 30 s break was included. To familiarize participants with color-button arrangements and thereby reducing eye-movements during the task, subjects were run on 40 practice trials with colored squares before block one and on an additional 12 practice trials before block two to four, respectively. Stimulus presentation and response recording were controlled by Presentation Software 21.1 (Neurobehavioral Systems Inc., Albany, CA, United States) run on a Pentium (Intel Corp., Santa Clara, CA, United States)-based personal computer. Depending on the subjects’ speed, the EPSP took approximately 20 min (including breaks and practice trials).

### Event-Related Potentials Recording and Quantification

Throughout the EPSP, EEG was continuously recorded from 64 active (Ag/AgCl) electrodes mounted in an elastic cap (actiCap snap, Easycap GmbH, Herrsching, Germany). Electrode sites were re-referenced online to FCz. Scalp impedances were kept below 20 kΩ. Signals were recorded using Brain Vision Recording Software (Version 1.22.0101) and a BrainAmp DC amplifier (both Brain Products GmbH, Gilching, Germany) with a sampling rate of 500 Hz and a band-pass filter between 0.1 and 80 Hz. Brain Vision Analyzer software 2.2.0 (Brain Products GmbH, Gilching, Germany) was used for offline processing. Raw EEG data was filtered with a 0.5 Hz (12 dB/oct per order) high-pass Butterworth IIR. Data was then visually inspected for non-ocular artifacts, and these were subsequently excluded. Blink- and eye- movement artifacts were removed using an Independent Component Analysis (ICA-) algorithm as implemented in Brain Vision Analyzer. Afterward, data was filtered using a 30 Hz (12 dB/oct per order) low-pass Butterworth IIR and a 50 Hz Notch filter, re-referenced to an average reference and segmented (stimulus locked; −200 ms to 1,000 ms). For each stimulus category, grand average waveforms were computed and baseline-corrected using the pre-stimulus interval (−200 ms to 0 ms). Three subjects (2 OC and 1 NC) had to be excluded from further analyses due to extensive EEG artifacts caused by movements, muscle tension or teeth grinding. For the remaining subjects, an average of 59.75 (*SD* = 3.25) trials per stimulus subcategory were included in the analysis. Symmetrical clusters were chosen for analysis of both ERP components. In accordance with its typical temporo-occipital topography, mean amplitudes of electrodes PO7, PO8, O1, and O2 in the time window between 150 and 250 ms were extracted to quantify the EPN component. For analyses of the LPP, mean amplitudes in the temporal window between 400 and 800 ms were extracted at centro-parietal electrodes CP1, CP2, P1, and P2. For both ERP components, choice of time window and electrodes was based on visual inspection of grand average waveforms, significant electrode inter-correlations and results of previous research (Farkas et al., 2020; Schindler et al., 2020).

### Endocrine Analyses

Prior to EEG recording, saliva samples for analysis of progesterone (P), 17β-estradiol (E2) and testosterone (T) were collected using Salicaps (IBL, Hamburg, Germany). These were stored in a freezer at −20°C before being analyzed using enzyme-linked-immunosorbent assay (ELISA-) kits (IBL Hamburg, Germany) in a fully automated manner (BEP2000, Siemens Healthineers, Eschborn, Germany). Only P and E2 were analyzed for the current study. All analyses were run in duplicates. Intra-assay variability coefficients were 7.5 and 7.8% for E2 and P, respectively. Inter-assay variability coefficients were 6.3% for E2 and 1.3% for P. The standard curves were of expected slope and shape and according to the manufacturer, all ELISA-kits were validated by LC-MS. Subjects were instructed to neither eat nor drink (except water) for 2 h prior to testing to avoid possible contamination of samples. Outliers were replaced using a 90% winsorization for the NC and OC groups, respectively. Consequently, values below the 5th or above the 95th percentile were replaced with values corresponding to the 5th or 95th percentile, respectively. Winsorization has proven as a reliable method for outlier correction (Kennedy et al., 1992; Rivest, 1994).

### Subjective Stimulus Evaluations

During their last appointment, subjects rated valence and arousal of the EPSP pictures on a 9-point rating scale (arousal: 1 calm and relaxed, 9 very aroused; valence: 1 very unpleasant, and 9 very pleasant) using Self-Assessment Manikins (SAM) (Bradley and Lang, 1994). Subjects were simultaneously presented with a printed version of all pictures belonging to the same subcategory (erotic: couple, male, female; neutral: couple, person, tree; positive). Presentation order of categories was randomized in order to avoid sequence effects. Stimulus evaluations were collected after the last EEG-recording to reduce the influence of conscious stimulus evaluations on neural processing.

### Statistical Analyses

Mean concentration of ovarian steroids was compared between groups using independent sample *t*-tests for (a) estradiol and (b) progesterone concentration, respectively. Differences between MC/OC phases were analyzed using rmANOVAs separately for each group. For analysis of the subjective stimulus evaluations, arousal and valence ratings were entered into two separate rmANOVAs with the within-subjects factor stimulus category (three steps) and the between-subjects factor group (two steps). Since OC/MC phase of stimulus evaluation was not balanced, this was also added as a between-subjects factor.

To compare EPN and LPP amplitudes between groups, ERP amplitudes were averaged across measurement times. They were then entered into two rmANOVAs with the within-subjects factors electrode (four steps) and stimulus category (three steps) and the between-subjects factor group (two steps). To assess OC-regimen related effects, ERP amplitudes were then compared between measurement times across the OC regimen using rmANOVAs with the within-subjects factors electrode (four steps), measurement time (three steps) and stimulus category (three steps). Statistical analyses were conducted using IBM SPSS Statistics version 27 (IBM Corp., Somers, NY, United States) with an α-level set to 0.05. For all rmANOVAs Greenhouse–Geisser correction was used in case of violated sphericity assumption. Bonferroni correction was applied to control for multiple testing in *post hoc* analyses.

## Results

### Participants

The final sample consisted of 62 subjects (29 NC and 33 OC). Groups did not differ in age, BMI, age at menarche or relationship status (Table 1). Age and duration of OC use were unrelated, *r* = 0.266, *p* = 0.134, but age was positively correlated with duration of the hormone free interval in previous OC users, *r* = 0.723, *p* < 0.001.

**TABLE 1:** Participant characteristics of naturally cycling (NC) women and oral contraceptive (OC) users.

	Entire sample	NC	OC	Test-statistics
Age (mean ± *SD*)	23.24 ± 2.83	23.72 ± 2.56	22.82 ± 3.03	*t*_(60)_ = 1.26, *p* = 0.212
BMI (mean ± *SD*)	21.26 ± 1.87	21.37 ± 2.21	21.17 ± 1.55	*t*_(60)_ = 0.42, *p* = 0.676
Age at menarche (mean ± *SD*)	12.98 ± 1.75	12.62 ± 1.24	13.30 ± 2.07	*t*_(60)_ = 1.55, *p* = 0.127
Relationship status (% in a relationship)	75.80	72.40	78.80	χ^2^_(1)_ = 0.34, *p* = 0.559

### Gonadal Steroid Concentration

Mean estradiol (Table 2), *t*_(60)_ = 2.02, *p* = 0.048, *d* = 0.514, as well as progesterone concentration (Table 3), *t*_(60)_ = 4.12, *p* < 0.001, *d* = 1.049, differed significantly between groups, with, respectively, higher concentrations in the NC compared to the OC group. For the NC group, estradiol concentration (Table 2) differed significantly between MC phases, *F*_(2_,_56)_ = 6.85, *p* = 0.002, $\eta_{\text{p}}^{2}$ = 0.197. Pairwise comparison revealed that estradiol levels were significantly lower during FO compared to OV, *p* = 0.010, and LU, *p* = 0.015. No difference was observed between OV and LU, *p* = 1.00.

**TABLE 2:** Mean (M), standard deviation (SD) and range of estradiol concentration in pg/ml across follicular (FO), ovulation (OV) and luteal phase (LU) in naturally cycling (NC) women and across active OC pill phase one (AP1), active OC pill phase two (AP2), and the inactive OC pill phase (lP) in oral contraceptive (OC) users.

NC				
	**FO**	**OV**	**LU**	**Mean**
*M*	4.15	5.06	4.84	4.68
*SD*	0.94	1.28	1.37	0.91
Range	2.94–6.53	2.69–7.96	2.70–9.15	2.86–7.25

**OC**				

	**AP1**	**AP2**	**IP**	**Mean**
*M*	3.98	3.99	4.47	4.15
*SD*	1.08	1.40	1.62	1.15
Range	2.38–6.05	2.15–7.91	2.54–11.41	2.37–6.72

**TABLE 3:** Mean (M), standard deviation (SD) and range of progesterone concentration in pg/ml across follicular (FO), ovulation (OV) and luteal phase (LU) in naturally cycling (NC) women and across active OC pill phase one (AP1), active OC pill phase two (AP2) and the inactive OC pill phase (lP) in oral contraceptive (OC) users.

NC				
	**FO**	**OV**	**LU**	**Mean**
*M*	43.22	68.82	106.79	72.94
*SD*	25.76	101.92	55.16	45.31
Range	22.08–119.21	12.21–578.60	38.69–239.08	28.64–278.48

**OC**				

	**AP1**	**AP2**	**IP**	**Mean**
*M*	35.99	35.74	41.29	37.67
*SD*	12.70	18.62	37.61	17.96
Range	19.03–75.33	18.75–102.55	14.49–190.26	19.31–104.37

Progesterone concentration also differed significantly between MC phases, *F*_(1_._58_,_44_._36)_ = 7.48, *p* = 0.003, $\eta_{\text{p}}^{2}$ = 0.211, with significantly higher concentrations during LU compared to FO, *p* < 0.001, and no significant differences in FO and LU compared to OV (all *p* ≥ 0.119) as illustrated in Table 3.

As expected, no phase differences in gonadal steroid concentration were observed for the OC group, estradiol (Table 2): *F*_(1_._39_,_44_._53)_ = 2.85, *p* = 0.086, $\eta_{\text{p}}^{2}$ = 0.082; progesterone (Table 3): *F*_(1_._52_,_48_._68)_ = 0.68, *p* = 0.473, $\eta_{\text{p}}^{2}$ = 0.021.

### Subjective Stimulus Evaluations

#### Arousal Ratings

**FIGURE 3:**
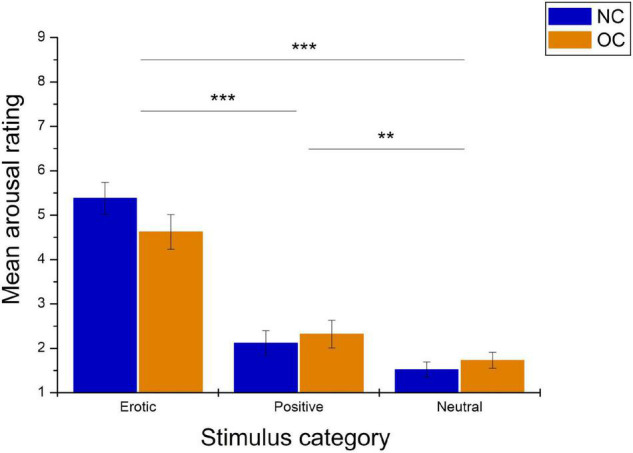
Mean arousal ratings in reaction to erotic, positive and neutral stimuli in naturally cycling (NC) women and oral contraceptive (OC) users. Error bars reflect standard error of the mean (SEM). ***p < 0.001, **p < 0.01.

Arousal ratings differed significantly between stimulus categories, *F*_(1_._66_,_93_._04)_ = 126.72, *p* < 0.001, $\eta_{\text{p}}^{2}$ = 0.694 and were not affected by group, *F*_(1_,_56)_ = 0.13, *p* = 0.720, $\eta_{\text{p}}^{2}$ = 0.002. The stimulus category × group interaction was also not significant, *F*_(1_._66_,_93_._04)_ = 3.04, *p* = 0.062, $\eta_{\text{p}}^{2}$ = 0.052. Pairwise comparison revealed significantly higher arousal ratings in reaction to erotic vs. neutral and positive stimuli (all *p* < 0.001). Positive stimuli were rated as more arousing compared to neutral stimuli, *p* = 0.004. See Figure 3 for illustration.

#### Valence Ratings

**FIGURE 4:**
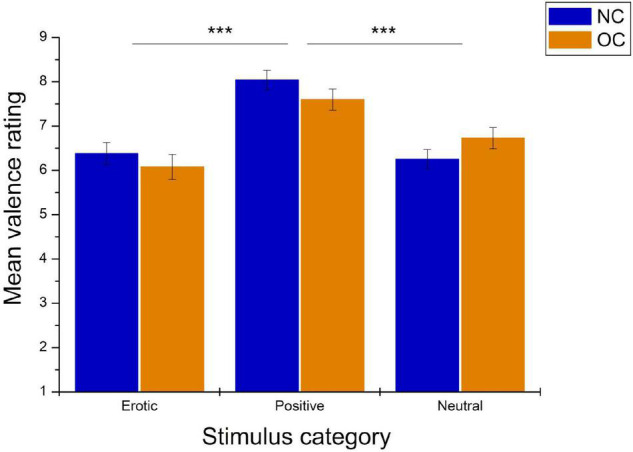
Mean valence ratings in reaction to erotic, positive and neutral stimuli in NC women and OC users. Error bars reflect SEM. ***p < 0.001.

As shown in Figure 4, valence ratings also differed significantly between stimulus categories, *F*_(1_._78_,_99_._45)_ = 29.08, *p* < 0.001, $\eta_{\text{p}}^{2}$ = 0.342. Pairwise comparison revealed that positive stimuli were rated as significantly more pleasant compared to erotic and neutral stimuli (all *p* < 0.001), while no differences were observed between the latter two categories, *p* = 0.750. Valence ratings did not differ between groups, *F*_(1_,_56)_ = 0.15, *p* = 0.704, $\eta_{\text{p}}^{2}$ = 0.003, nor was there a significant stimulus category × group interaction, *F*_(1_._78_,_99_._45)_ = 2.50, *p* = 0.094, $\eta_{\text{p}}^{2}$ = 0.043.

### Neural Reactivity

#### Early Posterior Negativity

**FIGURE 5:**
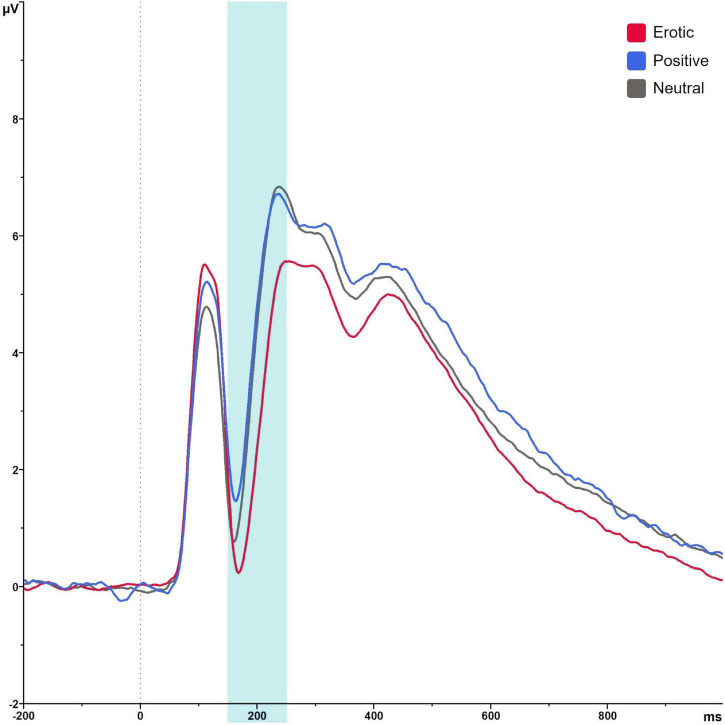
Grand average on electrode PO7 in reaction to erotic, positive and neutral stimuli in the time window 150–250 ms.

Regarding the EPN, analyses revealed a main effect of stimulus category, *F*_(1_._37_,_82_._23)_ = 70.79, *p* < 0.001, $\eta_{\text{p}}^{2}$ = 0.541. Pairwise comparison showed that erotic stimuli evoked greater EPN negativity compared to neutral and positive stimuli (all *p* < 0.001). No differences were observed between positive and neutral stimuli, *p* = 0.061. Mean amplitude was 3.31 μV (*SEM* = 0.41) in reaction to erotic, 4.89 μV (*SEM* = 0.38) in reaction to positive and 4.69 μV (*SEM* = 0.38) in reaction to neutral stimuli (Figure 5).

**FIGURE 6:**
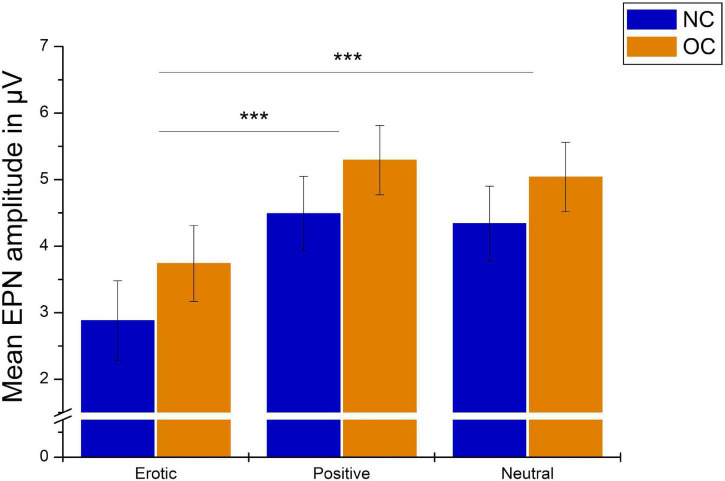
Mean EPN amplitudes in μV averaged over electrodes PO7, PO8, O1, and O2 in reaction to erotic, positive and neutral stimuli in NC women and OC users. Error bars reflect SEM. ***p < 0.001.

Groups did neither differ significantly in EPN amplitudes, *F*_(1_,_60)_ = 1.05, *p* = 0.311, $\eta_{\text{p}}^{2}$ = 0.017, nor was there a significant group × stimulus category interaction, *F*_(1_._37_,_82_._23)_ = 0.14, *p* = 0.869, $\eta_{\text{p}}^{2}$ = 0.002. Results are illustrated in Figure 6. EPN amplitudes did not differ in dependence of OC regimen, *F*_(2_,_64)_ = 1.27, *p* = 0.288, $\eta_{\text{p}}^{2}$ = 0.038 and there was no significant OC regimen × stimulus category interaction, *F*_(4_,_128)_ = 1.48, *p* = 0.213, $\eta_{\text{p}}^{2}$ = 0.044.

#### Late Positive Potential

**FIGURE 7:**
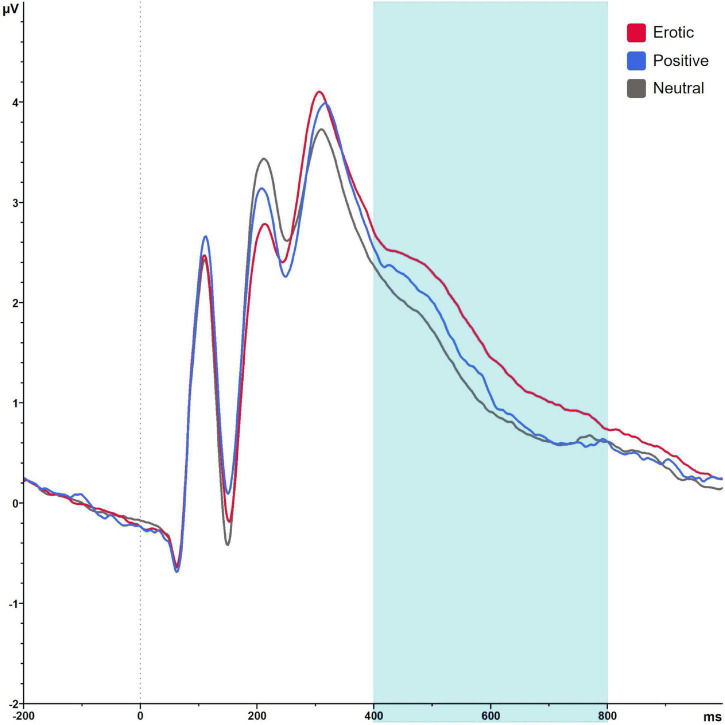
Grand average on electrode P2 in reaction to erotic, positive and neutral stimuli in the time window 400–800 ms.

Late positive potential amplitudes differed significantly in dependency of stimulus category, *F*_(1_._69_,_101_._36)_ = 67.87, *p* < 0.001, $\eta_{\text{p}}^{2}$ = 0.531. Erotic stimuli elicited greater LPP amplitudes compared to neutral and positive ones (all *p* < 0.001). LPP amplitudes toward positive vs. neutral stimuli did not differ significantly, *p* = 1.00 (Figure 7).

**FIGURE 8:**
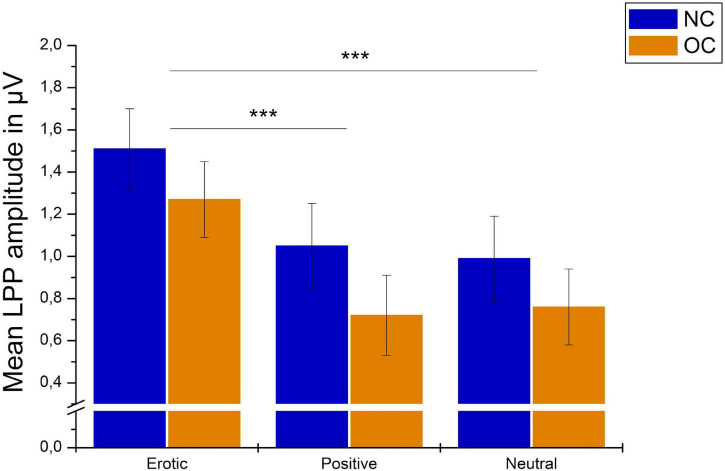
Mean LPP amplitudes in μV averaged over electrodes CP1, CP2, P1, and P2 in reaction to erotic, positive and neutral stimuli in NC women and OC users. Error bars reflect SEM. ***p < 0.001.

There was no significant main effect of group, *F*_(1_,_60)_ = 1.04, *p* = 0.311, $\eta_{\text{p}}^{2}$ = 0.017, and no significant group × stimulus interaction, *F*_(1_._69_,_101_._36)_ = 0.53, *p* = 0.560, $\eta_{\text{p}}^{2}$ = 0.531, $\eta_{\text{p}}^{2}$ = 0.009. Results are illustrated in Figure 8.

**FIGURE 9:**
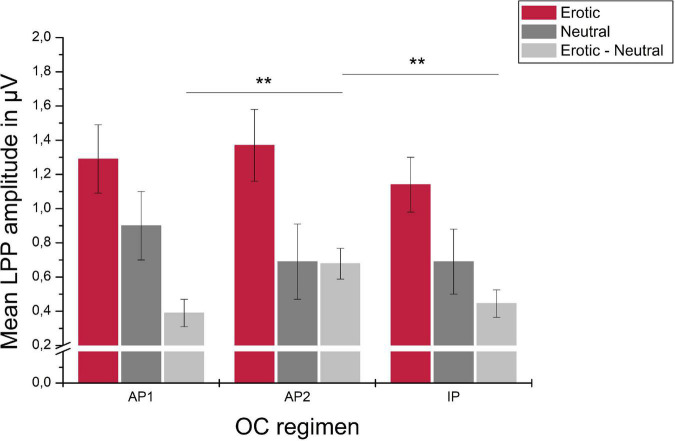
Mean LPP amplitudes in μV averaged over electrodes CP1, CP2, P1, and P2 in reaction to erotic and neutral stimuli as well as the erotic – neutral difference in OC users across active OC pill phase one (AP1), active OC pill phase two (AP2) and the inactive OC pill phase (lP). Error bars reflect SEM. **p < 0.01.

For the OC group, LPP amplitudes differed across the OC regimen in dependency of stimulus category, as indicated by a significant stimulus × OC regimen interaction, *F*_(3_._07_,_98_._07)_ = 3.85, *p* = 0.011, $\eta_{\text{p}}^{2}$ = 0.107. *Post hoc* analyses revealed no main effect of measurement time for neither stimulus category, erotic: *F*_(2_,_64)_ = 1.38, *p* = 0.259; neutral: *F*_(2_,_64)_ = 1.59, *p* = 0.212; positive: *F*_(2_,_64)_ = 0.14, *p* = 0.872. The amplitude difference between erotic and neutral stimuli, however, differed significantly across OC regimen measurement times, *F*_(2_,_64)_ = 9.58, *p* < 0.001, $\eta_{\text{p}}^{2}$ = 0.230, and was higher during AP2 compared to AP1 and IP, *p* = 0.001, respectively. Descriptively, this effect was due to lower reactivity toward neutral stimuli (AP1 vs. AP2) and higher neural reactivity toward erotic stimuli (AP1 vs. AP2 and AP2 vs. IP). Results are illustrated in Figure 9.

## Discussion

The current study examined neural correlates of early and later stages of emotional processing in NC and OC women using an ERP approach. NC women and OC users did not differ significantly in neural reactivity toward positive or erotic visual stimuli, nor did they rate valence and arousal of these stimuli differently. In OC using women, LPP but not EPN amplitude differences between erotic and neutral stimuli varied significantly between measurement times across the OC regimen with significantly higher differences during AP2 compared to AP1 and IP – that is, during the second week of OC intake compared to the first and the OC free week.

### Gonadal Steroid Concentration

Endogenous estradiol as well as progesterone concentrations were lower in the OC than in the NC group. These results are in line with most previous studies (Rapkin et al., 2006; Petersen and Cahill, 2015; Zethraeus et al., 2017) and are caused by OCs downregulating effect on the HPG-axis. Yet, not all studies did report differences in estradiol levels between NC and OC subjects (Monciunskaite et al., 2019), possibly due to the use of between- vs. within-subjects designs. In the current study, reliability of endocrine analyses was increased by strict termination of measurements in three distinct cycle phases and the assessment of these distinct phases in the same women, which helped to reduce between-subject variance. If distinct phases are being assessed in different women, high-between subject variance can hinder detection of phase and/or group differences. Within-subject designs are, therefore, advantageous in order to disentangle these effects. In the NC group, endocrine concentrations showed expected variations across the menstrual cycle. Estradiol concentration was highest during ovulation and remained elevated during luteal compared to follicular phase. This corresponds to the second estrogen peak observed during mid-luteal phase (Gandara et al., 2007). Progesterone levels peaked during luteal phase with a significant increase compared to follicular phase and intermediate concentrations during ovulation.

### Subjective Stimulus Evaluations

Regarding stimulus arousal ratings, both emotional categories were rated as significantly more arousing compared to neutral stimuli and erotic stimuli were also rated as more arousing compared to positive stimuli. This is in line with previous research (Jacob et al., 2011; Maffei et al., 2015). With regard to valence ratings, positive stimuli were rated as significantly more pleasant than neutral and erotic stimuli. In contrast to our expectation, erotic stimuli were not rated as more pleasant than neutral stimuli. Higher pleasantness ratings for erotic vs. neutral stimuli have previously been reported (Jacob et al., 2011), but might depend on the explicity of erotic stimuli, perceived embarrassment while watching such stimuli, or specific features of included neutral stimuli (Maffei et al., 2015). Specific setting characteristics (online vs. on-site) could also affect the results with anonymous online ratings possibly being more honest. In the current study, no significant differences between the NC and OC group in valence or arousal ratings regarding any stimulus category were observed. Accordingly, Armbruster et al. (2017) reported no differences in subjective stimulus evaluations between NC and OC women in reaction to positive, negative and neutral stimuli. Subtle differences in valence and arousal ratings in reaction to positive, negative and neutral stimuli have previously been reported by Spalek et al. (2019). However, the authors did not control for measurement time during MC/OC regimen and their sample included various types of HCs (COCs, patches, hormonal IUDs, and progestin-only pills).

Regarding erotic stimuli, Abler et al. (2013) did not observe group differences in arousal ratings. The current results, while remaining on a trend level (*p* = 0.062 for the stimulus × group interaction), indicate slightly lower arousal ratings in OC users compared to NC women. This finding is relevant in the light of adverse sexual effects reported by some OC users (Wallwiener et al., 2010, 2015; Smith et al., 2014; Zethraeus et al., 2016; Huang et al., 2020) and should be more closely examined in future studies, possibly in combination with measures of sexual function and/or self-reported adverse OC effects.

Results suggest that conscious processing of emotional stimuli might not be influenced very strongly by OC intake. Previous results regarding modulation of subjective stimulus evaluations by MC phase were inconsistent with some studies reporting no (Zhang et al., 2013) and others domain-specific effects (Lusk et al., 2015; Mačiukaitė et al., 2017). To the best of our knowledge, no study so far assessed OC regimen related effects in stimulus evaluations. As MC/OC regimen time points of stimulus evaluation were not balanced between subjects in the current sample, no conclusion can be drawn in this respect.

### Neural Reactivity in Naturally Cycling vs. Oral Contraceptive Using Women

No significant differences were observed in neural reactivity between NC and OC women neither for early (EPN) nor for later (LPP) stages of emotional processing. These differences were expected due to reported adverse side effects of OC intake, including depression (Skovlund et al., 2017; Wit et al., 2020), a reduction of general well-being (Zethraeus et al., 2017) or sexual function (Wallwiener et al., 2010, 2015; Smith et al., 2014; Zethraeus et al., 2016), and previous research indicating reduced reactivity to negative emotional stimuli under OC treatment (Gingnell et al., 2013; Petersen and Cahill, 2015; Monciunskaite et al., 2019). However, Abler et al. (2013) also reported no significant differences in neural activity during viewing of erotic videos and pictures between OC using and NC women. They did observe subtle differences in activity of the precentral gyrus during expectation of erotic stimuli but only when comparing follicular NC women to OC users that had previously taken OC pills for up to 2 months without any OC break, which limits generalizability of reported results.

Generally, an important factor that needs to be considered in OC research and interpretation of its results is the duration of OC use. In case of the Gingnell et al. (2013) study, emotional processing was assessed after one cycle of OC treatment and only women who had previously experienced OC related side effects were included. Mean duration of OC intake in the current sample was approximately 4 years. Consequently, prolonged rather than initial effects of OC use were assessed. Initial effects of OC treatment – as assessed after one treatment cycle – might diminish over time leading to non-significant findings in cross-sectional studies. Correspondingly, Jarva and Oinonen (2007) reported greater differences between NC and OC women in emotional reactivity if those took OCs for less than 2 years. Exploratory analyses in the underlying sample revealed no significant association between duration of OC use and neural reactivity, neither in the EPN nor in the LPP. However, no subject in the current sample had used OCs for less than 12 months and only four had used them for less than 2 years. Consequently, no conclusions could be drawn on initial OC treatment effects.

Nevertheless, some effects of OCs on brain structure and neurochemistry might last for months or years after OC discontinuation (Pletzer et al., 2019) and such enduring effects could also mask group differences if NC women have previously used HCs. Importantly, 22 out of 29 subjects in the current NC sample reported previous HC use. Therefore, reported results might not be generalizable to HC naïve women. Adverse side effects are the main reason for terminating (oral) hormonal contraception (Sanders et al., 2001; Huber et al., 2006). The relatively long mean duration of OC use in the underlying OC group suggests an absence of major side effects. This phenomenon is called self-selection bias or survivor-effect and is highly relevant in cross-sectional OC research (Brønnick et al., 2020). Consequently, OC users in the current sample might not be representative of OC users in general, but of those without major side effects. These women might also experience only subtle alterations of psychophysiological processes. However, 42% of OC users in the current sample reported to experience some sort of OC related side effects with mood swings and depressive mood being most frequently reported. Practical reasons, partnership characteristics or a positive cost-benefit ratio (cycle control, beneficial effects on acne or menstrual pain) might result in continuation despite side effects (Frost and Darroch, 2008; Merkh et al., 2009; Egarter et al., 2013). To differentiate effects of initial, prolonged or previous OC use, future studies should include higher proportions of OC naïve women. As recent studies suggest higher vulnerability to OC related neurophysiological alterations and side effects during puberty (Anderl et al., 2020; Sharma et al., 2020a; Wit et al., 2020), time of OC initiation should also be considered. Furthermore, information about side effects and reasons for initiation (i.e., contraception vs. health-related reasons) should also be collected more thoroughly.

Current research suggests that adverse effects following OC use occur only in a subgroup of women (Schaffir, 2006; Burrows et al., 2012; Schaffir et al., 2016). Accordingly, Scheuringer et al. (2020) conducted a placebo-controlled RCT and assessed attentional biases and depressive symptoms after 3 months of OC intake. While they did not observe effects of OCs on depressive symptoms, or interference in a verbal emotional Stroop task, previous OC use and high trait anxiety significantly predicted depressive symptoms at the end of the trial. Genetic factors could also contribute to such susceptibility and might be similar to those predisposing women to experience other reproductive affective disorders like premenstrual dysphoric disorder, postpartum or perimenopausal depression (McEvoy et al., 2017; Bromberger and Epperson, 2018). Regarding sexual side effects, polymorphisms associated with female sexual function such as the estrogen-receptor α polymorphism (rs2234693) (Armeni et al., 2017) could be relevant as well. Such factors should be considered in future research to prospectively improve contraception counseling.

In a previous ERP study on OC use, Monciunskaite et al. (2019) reported significantly lower LPP amplitudes toward emotional as well as neutral stimuli in OC users. As the study design of this study was relatively similar (ERP approach, student sample, comparable sample size, and long OC duration), previously outlined factors are unlikely to account for diverging results. However, the sample in the Monciunskaite et al. (2019) study was restricted to users of anti-androgenic progestins. In the current sample, a majority of women used androgenic progestins. While most studies do not control for different OC formulations, some recent results indicate that androgenic vs. anti-androgenic progestins differentially affect cognitive and socio-emotional processes. For instance, Gurvich et al. (2020) reported lower accuracy in an emotional face discrimination paradigm in anti-androgenic vs. androgenic OC users. Pletzer et al. (2015) observed higher accuracy in a neutral face recognition paradigm and higher gray matter volumes in the fusiform gyrus, the fusiform face area and the parahippocampal place area as well as the cerebellum in users of anti-androgenic progestins. Face recognition accuracy did not differ significantly between NC women and users of androgenic progestins. In a later study, Pletzer et al. (2016) reported that observed differences between NC and OC women in resting-state-connectivity were mostly attributable to the group of anti-androgenic OC users. Affected regions belonged to limbic as well as occipital networks involved in processing of visual emotional stimuli. Exploratory analysis in the current sample revealed a trend (*p* = 0.052 for the main effect of group) toward lower LPP amplitudes in anti-androgenic OC users compared to NC women and users of androgenic OCs. EPN amplitudes did not differ significantly between groups. However, subjective stimulus evaluation differed significantly between groups. Irrespective of stimulus category, users of anti-androgenic OCs rated stimuli as significantly less pleasant compared to androgenic OC users, who did not differ from the NC group. Regarding stimulus arousal, erotic stimuli were rated as significantly less arousing in the anti-androgenic- compared to the androgenic OC as well as the NC group. Test statistics regarding these results are provided in the Supplementary Material. These results should be treated with caution due to the small sub-sample size (23 subjects used androgenic OCs and 10 subjects anti-androgenic OCs). Taken together with results described above, they nevertheless suggest that pooling different OC formulations might prevent detection of group differences between NC and OC women and differential effects of progestin types should be considered in future research. Research regarding effects of synthetic progestins on the CNS is still very sparse (Gruber and Huber, 2003) and most studies also did not include/differentiate newer progestins (Rapkin et al., 2006; Africander et al., 2011; Porcu et al., 2019). Therefore, as a first step, more basic neuroscientific research, examining differential effects of OC preparations on i.e., neurosteroid concentration and/or neuroanatomy, is needed.

Other possible explanations for the diverging results between the current and the Monciunskaite et al. (2019) sample are design and paradigm-related differences. In the current study, each subject was assessed three times across the MC/OC regimen [compared to once in Monciunskaite et al.’s (2019) sample]. Measurements during ovulation in the NC and during the OC free week in the OC group were included. Furthermore, Monciunskaite et al. (2019) used a passive viewing paradigm whereas an active task requiring subjects to react to presented stimuli was used in the current study. Previous research indicated higher mind wandering tendencies in OC users (Raymond et al., 2019) – a confound that should be more relevant in passive vs. active tasks and might explain reduced LPP amplitudes in the Monciunskaite et al. (2019) sample. Further research, possibly including eye-tracking measures, is necessary, especially since NC and OC women might differ in their attention to contextual features of presented (erotic) stimuli (Rupp and Wallen, 2007).

Regarding erotic picture processing, another factor should be considered in future research: Prause et al. (2015) observed lower LPP amplitudes in subjects reporting less than two sexual intercourse partners during the last 12 months compared to subjects reporting two or more partners. Importantly, this difference was more pronounced in less explicit sexual images. As erotic pictures in the current study included males and females in underwear (i.e., no naked genitals were depicted) and couples during “petting” rather than penetrative intercourse, they can be classified as rather less explicit. Hence, differences in sexual activity between both groups could also have confounded current results. Subjects did not report the number of current and/or previous sexual intercourse partners. Relationship status – as the most similar measure in the underlying study – was, however, not significantly associated with early or late neural reactivity.

While no significant differences occurred between the NC and OC group, the erotic-neutral difference in LPP amplitudes was modulated by measurement time during the OC regimen with significantly higher differences during the second week of active OC intake. Importantly, this effect remained significant when controlling for phase of first measurement. Most previous studies on OC use did not examine different time points across the OC regimen at all, or if they did, compared one time point during the active period and one time point during the OC free week in a between-subjects design. Within-subjects designs – as used in the current study - help to reduce variance in order to better disentangle OC regimen effects. The higher erotic-neutral difference at AP2 resulted from lower neural reactivity to neutral stimuli (AP1 vs. AP2) and higher neural reactivity to erotic stimuli (AP1 vs. AP2 and AP2 vs. IP). Mean amplitude in reaction to erotic stimuli was highest at AP2, although this effect was not statistically significant. Previous research reported higher coupling of brain regions relevant for emotion regulation during the active vs. inactive period of the OC regimen (Nasseri et al., 2020) and Radke and Derntl (2016) reported higher accuracy during the active OC treatment in an affective responsiveness task. Taken together, reported results suggest that emotional processes are modulated by the OC regimen, with possibly higher emotional abilities (higher emotional reactivity to erotic vs. neutral stimuli, better emotion regulation and affective state evaluation) during active vs. inactive periods of the OC regimen.

After initiation of a new OC blister, ovarian suppression is usually reached after seven consecutive days of OC intake (Curtis et al., 2006). Most manufacturers recommend additional contraception if a pill is forgotten during the first 7 days after the break, but not if it is forgotten on days 8–14. Missing pills adjacent to the OC free interval is associated with greater risk of pregnancy (Curtis et al., 2006). The AP1 measurement in the current study corresponded to days 7–9 after starting a new OC blister and the AP2 measurement was conducted on days 14–16. Therefore, AP2 might correspond to the time point of maximal ovarian suppression and most stable endocrine status. Correspondingly, higher reactivity to erotic vs. neutral stimuli was noted at this point. This could be interpreted in terms of positive mood effects during steady hormonal states in OC users (Ott et al., 2008). Lower reactivity to erotic vs. neutral stimuli during the OC free week is in line with reported adverse effects during the hormone absent interval of the OC regimen (Sulak, 2000; Kelly et al., 2010). In the current study, it was aimed to assess NC and OC women on similar days of the MC/OC regimen with equal between measurement intervals. To further elucidate OC regimen related differences in emotional processing, future studies should terminate measurements in the OC group in consideration of results regarding ovarian function at different time points of the OC regimen (Curtis et al., 2006).

### Limitations

Several limitations need to be considered in the interpretation of the presented results. Most subjects were students and mean age was comparably young. Strict inclusion/exclusion criteria, furthermore, reduces representativeness of the sample.

Regarding included stimulus categories, no significant differences were observed in neural reactivity to positive vs. neutral stimuli. This contradicts previous research (Schupp et al., 2000; Bublatzky et al., 2014; Munk et al., 2016). However, neural reactivity has proven to be arousal- rather than valence dependent (Schupp et al., 2000; Leite et al., 2012). Comparing arousal ratings between subcategories revealed that positive stimuli were rated as more arousing compared to trees but did not differ significantly from neutral persons and couples which could explain the observed similarity in neural reactivity.

### Conclusion

Sixty years after introduction of “the pill,” the question *how* OCs affect brain structure and functions remains unclear and understudied (Porcu et al., 2019). Hence, in a first step, more basic neuroscientific research is needed.

In the current study, no significant differences were observed either in subjective stimulus evaluations, or in neural reactivity toward positive or erotic emotional stimuli. Neural reactivity toward erotic vs. neutral stimuli was modulated by time point of the OC regimen with greater differences during the second week of the OC regimen. This could result from steady endocrine states and should be more closely examined in future studies. Future studies should control for duration of current and/or previous OC use and other possible confounding factors such as the number of sexual intercourse partners. Furthermore, it seems to be highly relevant to distinctively examine different OC formulations, as different progestins could have differential effects on neural and socio-emotional processes.

## Data Availability Statement

The original contributions presented in the study are included in the article/Supplementary Material, further inquiries can be directed to the corresponding author.

## Ethics Statement

The studies involving human participants were reviewed and approved by the Local Ethic Commission of the Faculty of Psychology and Sport Science at Justus-Liebig-University Gießen. The patients/participants provided their written informed consent to participate in this study.

## Author Contributions

NS, JH, and AM: conceptualization and study design. NS: data acquisition and investigation, formal analysis, visualization, and writing – original draft. NS and AM: writing – review and editing. AM: project administration. JH and AM: supervision and resources. All authors contributed to the article and approved the submitted version.

## Conflict of Interest

The authors declare that the research was conducted in the absence of any commercial or financial relationships that could be construed as a potential conflict of interest.

## Publisher’s Note

All claims expressed in this article are solely those of the authors and do not necessarily represent those of their affiliated organizations, or those of the publisher, the editors and the reviewers. Any product that may be evaluated in this article, or claim that may be made by its manufacturer, is not guaranteed or endorsed by the publisher.
