# Supplementary material for: Event-Related Potentials in Women on the Pill: Neural Correlates of Positive and Erotic Stimulus Processing in Oral Contraceptive Users
Source: Front Neurosci. 2022 Jan 4;15:798823. doi: 10.3389/fnins.2021.798823 (PMC8764149; doi:10.3389/fnins.2021.798823)
Supplement: Supplementary file 2 [file Table_1.docx]

Supplementary Material

# Formulations of Oral Contraceptives and number (N) of users in the current sample (n = 33)

| Synthetic estrogen | Progestin | N users |
| --- | --- | --- |
| 0.02 mg Ethinylestradiol | 0.1 mg Levonogestrel | 10 |
|  | 0.15 mg Desogestrel | 3 |
|  | 2 mg Chlormadinon acetat | 2 |
|  | 3 mg Drospirenon | 1 |
| 0.03 mg Ethinylestradiol | 0.125 mg Levonogestrel | 1 |
|  | 0.15 mg Levonogestrel | 8 |
|  | 0.15 mg Desogestrel | 1 |
|  | 2 mg Dienogest | 5 |
|  | 2 mg Chlormadinon acetat | 1 |
| 0.035 mg Ethinylestradiol | 2 mg Cyproteron acetat | 1 |

# Exploratory analysis: Differences between naturally cycling (NC) women and users of androgenic or anti-androgenic oral contraceptives (OCs)

Regarding stimulus arousal, there was a significant stimulus × group interaction, *F*_(3.22, 85.28)_ = 3.08, *p* = .029, η_p_^2^ = .104. Pairwise comparisons revealed that groups differed significantly in arousal ratings of erotic stimuli, *F*_(2, 61)_ = 4.04, *p* = .023, η_p_^2^ = .132, with significantly lower arousal ratings in the anti-androgenic than in the androgenic, *p* = .040, as well as in the NC group, *p* = .032. Groups did neither differ significantly in arousal ratings of neutral, *F*_(2,61)_ = 1.08, *p* = .347, nor positive stimuli, *F*_(2, 61)_ = 1.81, *p* = .174.

Furthermore, groups differed significantly in stimulus valance ratings, *F*_(2,53)_ = 3.37, *p* = .042, η_p_^2^ = .113. Post-hoc comparison revealed that users of anti-androgenic OCs rated stimuli as significantly less pleasant compared to users of androgenic OCs, *p* = .040, irrespective of stimulus category. NC women did not differ from any OC group (all *p* ≥ .131).

Concerning EPN amplitudes towards all stimulus categories (erotic, positive, neutral), there was neither a significant main effect of group, *F*_(2,59)_ = 0.80, *p* = .453, nor a significant stimulus × group interaction, *F*_(2.74, 80.82)_ = 0.14, *p* = .921.

LPP-reactions towards all stimulus categories tended to be different between groups, *F*_(2,59)_ = 3.10, *p* = .052. Post-hoc comparison indicated lower amplitudes in anti-androgenic vs. androgenic OC users, *p* = .084, and NC women, *p* = .065.
